# A Novel Method for the Micro-Clearance Measurement of a Precision Spherical Joint Based on a Spherical Differential Capacitive Sensor

**DOI:** 10.3390/s18103366

**Published:** 2018-10-09

**Authors:** Wen Wang, He Yang, Min Zhang, Zhanfeng Chen, Guang Shi, Keqing Lu, Kui Xiang, Bingfeng Ju

**Affiliations:** 1 School of Mechanical Engineering, Hangzhou Dianzi University, Hangzhou 310018, China; wangwn@hdu.edu.cn (W.W.); zhangmme@foxmail.com (M.Z.); czf@hdu.edu.cn (Z.C.); shiguang@hdu.edu.cn (G.S.); lkq@hdu.edu.cn (K.L.); 2 School of Mechanical Engineering, Zhejiang University, Hangzhou 310027, China; snowboy2001102@163.com (K.X.); mbfju@zju.edu.cn (B.J.); 3 State Key Lab of Fluid Power & Mechatronic Systems, Zhejiang University, Hangzhou 310027, China

**Keywords:** spherical joint, clearance measurement, eccentric displacement, capacitor

## Abstract

A spherical joint is a commonly used mechanical hinge with the advantages of compact structure and good flexibility, and it becomes a key component in many types of equipment, such as parallel mechanisms, industrial robots, and automobiles. Real-time detection of a precision spherical joint clearance is of great significance in analyzing the motion errors of mechanical systems and improving the transmission accuracy. This paper presents a novel method for the micro-clearance measurement with a spherical differential capacitive sensor (SDCS). First, the structure and layout of the spherical capacitive plates were designed according to the measuring principle of capacitive sensors with spacing variation. Then, the mathematical model for the spatial eccentric displacements of the ball and the differential capacitance was established. In addition, equipotential guard rings were used to attenuate the fringe effect on the measurement accuracy. Finally, a simulation with Ansoft Maxwell software was carried out to calculate the capacitance values of the spherical capacitors at different eccentric displacements. Simulation results indicated that the proposed method based on SDCS was feasible and effective for the micro-clearance measurement of the precision spherical joints with small eccentricity.

## 1. Introduction

Because of the advantages of compact structure, good flexibility, and high carrying capacity, precision spherical joints are widely used for three-degree-of-freedom (DOF) mechanical hinges in many types of equipment, such as parallel mechanisms, industrial robots, and automobiles [1]. In actual applications of spherical joints, the clearance between the ball and the socket inevitably exists, due to manufacturing or installation errors. As a result, during the process of transmitting motion or force with spherical joints, the actual position and orientation of the output shaft may deviate from the ideal pose, which affects the overall transmission accuracy of the system. Therefore, real-time detection of a precision spherical joint clearance is of great significance in analyzing the motion errors of the application systems (e.g., robot joints) and improving the transmission accuracy [2].

The clearance measurement is essentially the detection of the relative linear displacement between two components. In the case of a precision spherical joint, the clearance measurement can be achieved by detecting the eccentric displacements of a ball (3-DOF motion in space) and the socket (fixed in space). Many methods have been proposed for the clearance measurement in industrial applications, for example, the inductive sensor-based method [3], the optical fiber-based method [4], the microwave-based method [5], and other methods based on eddy current sensors [6] or capacitive sensors [7]. However, studies focused on the clearance measurement of a precision spherical joint have been rarely reported. Recently, Endemano et al. [8] presented a two-dimensional (2D) study for calculating the capacitances of rotating objects with eccentricity. Han et al. [9] carried out a micro-displacement detection of a spherical rotor with regard to electrostatically suspended gyroscope technology. In the electrostatically suspended gyroscope’s rotor-bearing system, the spherical rotor was steadily suspended by electrostatic forces in a high vacuum chamber. The drift of the spherical rotor from the center of the cavity was obtained by detecting the variation of the spherical capacitance between electrodes and the rotor. This real-time drift was then used as a feedback signal to generate the feedback control of the electrostatic force. The spherical detecting electrode usually consisted of several electrodes (e.g., six, eight or twelve electrodes) [10,11,12,13,14]. Since the clearance of a spherical joint exhibits similar characteristics with that of a spherical rotor, this micro-displacement detection method used for the spherical rotor may provide an inspiration for spherical gap measurement.

A capacitive sensor is widely used for high-precision displacement detection in many applications [15,16]. It is a typical non-contact measurement device with high accuracy, good dynamic performance, high resolution, and strong adaptability to harsh environments (e.g., high temperature). Thus, in this paper, a spherical differential capacitive sensor (SDCS) is proposed to measure spatially eccentric displacements of the ball of a precision spherical joint. First, the structure and measuring principle of the SDCS is introduced. Then, the mathematical model for the relation between spatial eccentric displacements and differential capacitances is presented. Finally, the feasibility of the proposed method is validated by calculating the capacitance for different eccentric displacements using commercial software Ansoft Maxwell 16.0 (Ansoft, Pittsburgh, PA, USA).

## 2. Structural Design and Measuring Principle

**Figure 1:**
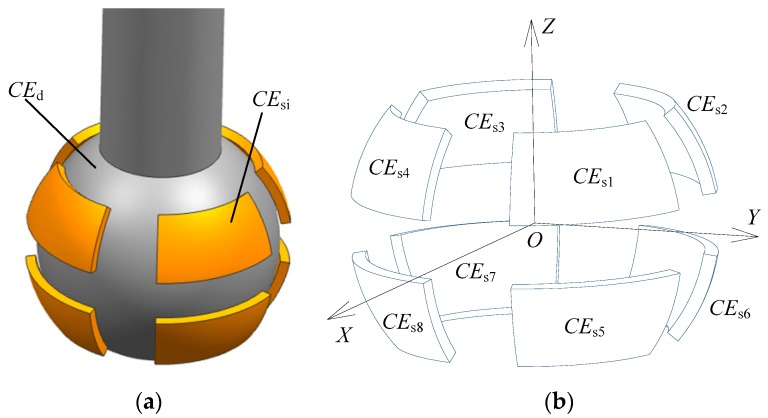
Structural model of the proposed SDCS: (**a**) assembly diagram and (**b**) distribution of the spherical capacitive plates.

The SDCS proposed in this work consists of a ball (excitation electrode, *CE*_d_) and eight spherical capacitive plates (sensing electrodes, *CE*_s*i*_, *i* = 1, 2, …, 8), as shown in Figure 1a. The ball of the spherical joint is used as a common electrode, whereas each of the eight spherical capacitive plates is employed as a spherical capacitor *C_i_*, *i* = 1, 2, …, 8. The coordinate system *OXYZ* is defined in Figure 1b, with the origin *O* chosen at the center of the spherical capacitive plates. Eight spherical capacitive plates having the same structure size are concentrically fixed in the spherical joint’s socket, and they are symmetrically distributed in eight quadrants of the three-dimensional coordinate system (Figure 1b). Teflon-laced epoxy with a certain thickness, known as an abrasion-resistant material with self-lubricating properties, was deposited on the inner surface of the spherical capacitive plates. This ensured the insulation between the excitation electrode and the sensing electrodes. Moreover, as a dielectric material, Teflon-laced epoxy can improve the sensitivity of the capacitive sensor.

Ideally, the center of the ball coincides with that of the spherical capacitive plates, and the clearances between the ball and each of the eight spherical capacitive plates have identical values. As such, eight spherical capacitors have the same value in capacitance. Once the ball has an eccentric displacement relative to the socket, the clearances between the eight spherical capacitive plates and the ball exhibit diverse variations, and thus the capacitance value of each spherical capacitor varies accordingly. In other words, the eccentric displacements of the ball can be obtained by detecting the capacitance of the eight spherical capacitors.

To obtain the micro-clearance of a precision spherical joint (i.e., the three-dimensional displacement of the ball), three pairs of differential capacitance units were established for the eccentric displacement measurement along the *X*, *Y* and *Z* directions, respectively. The differential capacitance unit pair denoted a pair of differential capacitance units that faced each other with a 180° phase shift. As shown in Table 1, *C*_up-*X*_, *C*_up-*Y*_ and *C*_up-*Z*_ denoted the differential capacitance unit pairs along the *X*, *Y* and *Z* directions, respectively. Each pair consisted of unit 1 and unit 2, and each unit included four adjacent capacitors. The eccentric displacements of the ball were calculated from the capacitance values of each differential capacitance unit pair. Specifically, the eccentric displacement *δ*_X_ of the ball along the *X* direction was obtained by calculating the capacitance difference between the two differential capacitance units along the *X* direction, one being the unit that consisted of *C*_1_, *C*_4_, *C*_5_ and *C*_8_, and the other being the unit that consisted of *C*_2_, *C*_3_, *C*_6_ and *C*_7_. Similarly, the eccentric displacement *δ*_Y_ of the ball along the *Y* direction and the eccentric displacement *δ*_Z_ of the ball along the *Z* direction was acquired by calculating the capacitance difference of the corresponding differential capacitance unit pair, respectively. Thus, the eccentric displacement component *δ*_X_, *δ*_Y_ and *δ*_Z_ of the ball can be given by the following equations:

(1) $$\delta_{X}=f_{X}(C_{1}+C_{4}+C_{5}+C_{8}-C_{2}-C_{3}-C_{6}-C_{7}),$$

(2) $$\delta_{Y}=f_{Y}(C_{1}+C_{2}+C_{5}+C_{6}-C_{3}-C_{4}-C_{7}-C_{8}),$$

(3) $$\delta_{Z}=f_{Z}(C_{1}+C_{2}+C_{3}+C_{4}-C_{5}-C_{6}-C_{7}-C_{8}),$$

where *f*_X_, *f*_Y_ and *f*_Z_ denote the transition function for the measured capacitance and the eccentric displacement component in the *X*, *Y* and *Z* directions, respectively. Their values were determined by the structural parameters of the eight spherical capacitors. In order to simplify the expressions of the differential capacitances along the *X*, *Y* and *Z* directions, Δ*C*_X_, Δ*C*_Y_ and Δ*C*_Z_ are defined as follows:

(4) $$\Delta C_{X}=C_{1}+C_{4}+C_{5}+C_{8}-C_{2}-C_{3}-C_{6}-C_{7},$$

(5) $$\Delta C_{Y}=C_{1}+C_{2}+C_{5}+C_{6}-C_{3}-C_{4}-C_{7}-C_{8},$$

(6) $$\Delta C_{Z}=C_{1}+C_{2}+C_{3}+C_{4}-C_{5}-C_{6}-C_{7}-C_{8}.$$

To approximately calculate the capacitance value of each spherical capacitor, two assumptions were made: (1) the area element d*A* of a spherical capacitor was regarded as a parallel-plate capacitor with an equal gap; (2) the fringe effect of the spherical capacitive plates was not taken into consideration. Based on the above assumptions, the capacitance of the spherical capacitor element d*C* was calculated based on the formula of a parallel-plate capacitor. Then, the capacitance value of each spherical capacitor was obtained using the following integral:

(7) $$C_{i}=\varepsilon \iint_{A_{i}}\frac{1}{e}dA\;(i=1,2,\cdots ,8),$$

where *ε* is the permittivity of the dielectric which includes the abrasion-resistant material and a small quantity of air, *e* is the clearance between the ball and each spherical capacitive plate, and *A_i_* is the effective area of the spherical capacitive plate for each spherical capacitor *C_i_*, *i* = 1, 2,…, 8.

## 3. Mathematical Model

### 3.1. Calculation of the Clearance between the Ball and the Spherical Capacitive Plates

**Figure 2:**
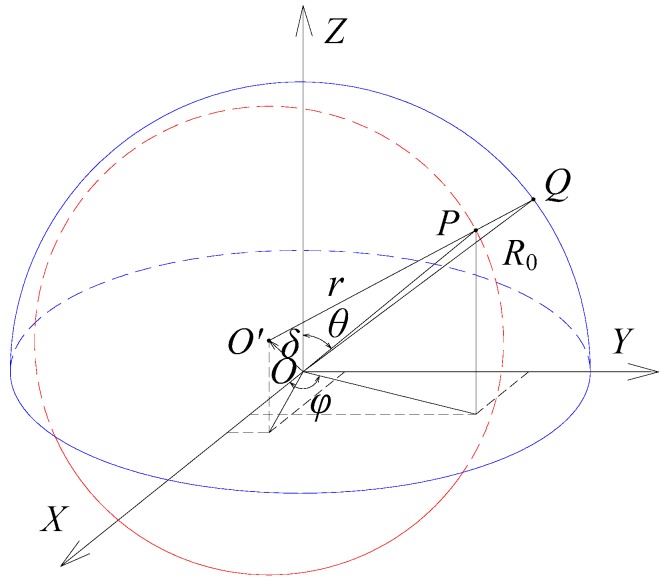
Schematic diagram of the spherical clearance calculation.

Figure 2 presents the coordinate system *OXYZ* in which the center of the ball deviates from that of the spherical capacitive plates. The unit vectors along the *X*, *Y* and *Z* directions are denoted by **i**, **j** and **k**, respectively. *θ* is the zenithal angle between the output shaft central axis of the spherical joint and the positive direction of the *Z* axis, 0≤θ≤π. *φ* is the azimuthal angle between the projection of the output shaft central axis onto the plane *XOY* and the positive direction of the *X* axis, 0≤φ≤2π. The point *P* is on the surface of the ball, and the outward unit normal vector **n** of the vector **OP** in the coordinate system *OXYZ* is given by the following equation:

(8) n=sinθcosφi+sinθsinφj+cosθk.

The inner surface of the spherical capacitive plates and the outer surface of the ball are considered as ideal spheres. As shown in Figure 2, *R*_0_ denotes a spherical radius of the inner surface of the spherical capacitive plates. *r* is the radius of the ball. *δ* is the linear displacement between the center *O* of the socket and the center *O*’ of the ball. Therefore, the spatially eccentric displacement of the ball can be written in the form of a vector as follows:

(9) $$\delta =\delta_{X}i+\delta_{Y}j+\delta_{Z}k.$$

As shown in Figure 2, the line joining the two points *O’* and *P* is extended to intersect with the inner surface of the spherical capacitive plates at the point *Q*. The distance from point *Q* to point *P* is defined as the clearance *e* at the point *P*. The initial clearance of the ball without eccentricity is denoted by *e*_0_, that is, $e_{0}=R_{0}-r$. If the ball has an eccentric displacement relative to the socket, the clearance at the point *P* in the case of $e\ll R_{0}$ can be given by the following equation:

(10) $$e=R_{0}-\delta_{X}\sin\theta \cos\varphi -\delta_{Y}\sin\theta \sin\varphi -\delta_{Z}\cos\theta -r.$$

To simplify the subsequent calculations, dimensionless parameters are introduced as follows:

$$k=\frac{e}{e_{0}},\lambda_{X}=\frac{\delta_{X}}{e_{0}},\lambda_{Y}=\frac{\delta_{Y}}{e_{0}},\lambda_{Z}=\frac{\delta_{Z}}{e_{0}}.$$

Therefore, Equation (10) can be rewritten in the following dimensionless form:

(11) $$k=1-\lambda_{X}\sin\theta \cos\varphi -\lambda_{Y}\sin\theta \sin\varphi -\lambda_{Z}\cos\theta .$$

### 3.2. Relation between Capacitance of Spherical Capacitors and Eccentric Displacements

Let $\lambda =\lambda_{X}\sin\theta \cos\varphi +\lambda_{Y}\sin\theta \sin\varphi$, assuming obviously that *λ* < 1. It is also assumed that the eccentric displacement of the ball is small.

Using Taylor’s expansion method for 1/*k*, it becomes the following Taylor’s series:

(12) $$\frac{1}{k}=\frac{1}{1-\lambda }=1+\lambda +\lambda^{2}+\lambda^{3}+\cdots .$$

Substituting Equations (11) and (12) into Equation (7), the following equation is obtained:

(13) $$C_{i}=\frac{\varepsilon }{e_{0}}\iint_{A_{i}}\left[1+E(\theta ,\varphi )\right]dA\;(i=1,2,\cdots ,8),$$

where $E(\theta ,\varphi )=\sum_{n=1}^{\infty }{\left(\lambda_{X}\sin\theta \cos\varphi +\lambda_{Y}\sin\theta \sin\varphi +\lambda_{Z}\cos\theta \right)}^{n}$ and d*A* is the area element of the spherical capacitive plates at the point *Q* (i.e., $dA=R_{0}{}^{2}\sin\theta d\theta d\varphi$).

The capacitance of each spherical capacitor can be obtained according to Equation (13). Then, the differential capacitance Δ*C*_X_, Δ*C*_Y_ and Δ*C*_Z_ can be calculated by substituting the capacitance of each spherical capacitor into Equations (4)–(6), respectively. Therefore, the mathematical model of the relation between the differential capacitance and the spatial eccentric displacements can be established.

Equation (13) can be further rewritten as follows:

(14) $$C_{i}=K_{i}(\lambda_{X},\lambda_{Y},\lambda_{Z})\frac{\varepsilon R_{0}{}^{2}}{e_{0}}\;(i=1,2,\cdots ,8),$$

where $K_{i}(\lambda_{X},\lambda_{Y},\lambda_{Z})=\iint_{A_{i}}[1+E(\theta ,\varphi )]\sin\theta d\theta d\varphi$.

**Figure 3:**
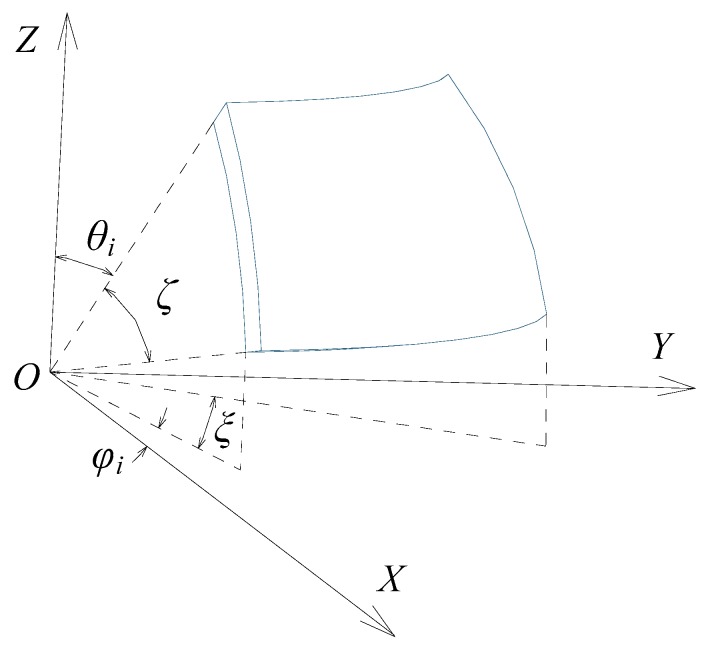
Coordinate system with a spherical capacitive plate *CE*_s1_.

As for the spherical capacitive plate *CE*_s*i*_ (*i* = 1, 2, …, 8) shown in Figure 3, ζ is the corresponding angle of arc length in the longitude direction, and $\theta_{i}$ denotes the initial zenithal angle between the upper edge of the spherical capacitive plate and the positive direction of the *Z* axis. ξ is the corresponding angle of arc length in the latitude direction, and $\varphi_{i}$ denotes the azimuthal angle between the projection of the left edge of the spherical capacitive plate onto the plane *XOY* and the positive direction of the *X* axis. Accordingly, $K_{i}(\lambda_{X},\lambda_{Y},\lambda_{Z})$ can be expressed as follows:

(15) $$K_{i}(\lambda_{X},\lambda_{Y},\lambda_{Z})=\int_{\theta_{i}}^{\theta_{i}+\zeta }\int_{\varphi_{i}}^{\varphi_{i}+\xi }\left[1+E(\theta ,\varphi )\right]\sin\theta d\theta d\varphi .$$

*K_i_* (*λ*_X_, *λ*_Y_, *λ*_Z_) can be solved according to Equation (15), after the layout and size of the spherical capacitive plates are determined. Since the eight spherical capacitive plates have the same structure size and are symmetrically distributed in space, and if the size and layout of the spherical capacitive plate *CE*_s1_ is known, other spherical capacitive plates can be determined. Considering the height restrictions of the spherical joint top-cover, let $\theta_{1}=5\pi /18$, ζ=π/6, $\varphi_{1}=\pi /12$, and ξ=π/3. Taking the spherical capacitor *C*_1_ as an example, the corresponding $K_{1}(\lambda_{X},\lambda_{Y},\lambda_{Z})$ is calculated as follows by neglecting the terms of degree above 3:

(16) $$\begin{array}{l} K_{1}(\lambda_{X},\lambda_{Y},\lambda_{Z})=0.4913+0.2987\lambda_{X}+0.2987\lambda_{Y}+0.2005\lambda_{Z}+\ldots \\ \;\;0.2002\lambda_{X}{}^{2}+0.2002\lambda_{Y}{}^{2}+0.0909\lambda_{Z}{}^{2}+0.3311\lambda_{X}\lambda_{Y}+\ldots \\ \;\;0.2383\lambda_{X}\lambda_{Z}+0.2383\lambda_{Y}\lambda_{Z}+0.1434\lambda_{X}{}^{3}+0.1434\lambda_{Y}{}^{3}+\ldots \\ \;\;0.0445\lambda_{Z}{}^{3}+0.3073\lambda_{X}\lambda_{Y}{}^{2}+0.3073\lambda_{X}{}^{2}\lambda_{Y}+0.1588\lambda_{X}\lambda_{Z}{}^{2}+\ldots \\ \;\;0.2341\lambda_{X}{}^{2}\lambda_{Z}+0.1588\lambda_{Y}\lambda_{Z}{}^{2}+0.2341\lambda_{Y}{}^{2}\lambda_{Z}+0.3873\lambda_{X}\lambda_{Y}\lambda_{Z} \end{array}$$

Similarly, $K_{i}(\lambda_{X},\lambda_{Y},\lambda_{Z})\;(i=2,\ldots ,8)$ for the other spherical capacitors can be obtained. Then, the capacitance value for each capacitor can be calculated according to Equation (14). By further substituting each capacitance into Equations (4)–(6), the following equations are obtained:

(17) $$\Delta C_{X}=\frac{\varepsilon R_{0}{}^{2}}{e_{0}}\Delta K_{X},$$

(18) $$\Delta C_{Y}=\frac{\varepsilon R_{0}{}^{2}}{e_{0}}\Delta K_{Y},$$

(19) $$\Delta C_{Z}=\frac{\varepsilon R_{0}{}^{2}}{e_{0}}\Delta K_{Z},$$

where

(20) $$\Delta K_{X}=2.3896\lambda_{X}+1.1472\lambda_{X}{}^{3}+2.4584\lambda_{X}\lambda_{Y}{}^{2}+1.2704\lambda_{X}\lambda_{Z}{}^{2},$$

(21) $$\Delta K_{Y}=2.3896\lambda_{Y}+1.1472\lambda_{Y}{}^{3}+2.4584\lambda_{Y}\lambda_{X}{}^{2}+1.2704\lambda_{Y}\lambda_{Z}{}^{2},$$

(22) $$\Delta K_{Z}=1.6040\lambda_{Z}+0.3560\lambda_{Z}{}^{3}+1.8728\lambda_{Z}\lambda_{X}{}^{2}+1.8728\lambda_{Z}\lambda_{Y}{}^{2}.$$

It can be seen from Equations (20)–(22) that the even-order terms of dimensionless eccentric displacements of the ball in the socket are eliminated. This can be ascribed to the symmetrically spatial distribution of the eight spherical capacitive plates and the differential calculation for the capacitance of the spherical capacitors. Thus, the proposed method can improve the sensitivity of the sensor and effectively reduce nonlinear errors.

To examine the effect of the third-order terms, the dimensionless eccentric displacements of the ball with and without the third-order terms were compared in detail. Two cases were considered, one being the displacements only in each axis direction (*λ*_X_ = 0, *λ*_Y_ = 0 or *λ*_Z_ = 0), the other being the displacements in a non-axis direction (*λ*_X_ ≠ 0, *λ*_Y_ ≠ 0 and *λ*_Z_ ≠ 0).

**Figure 4:**
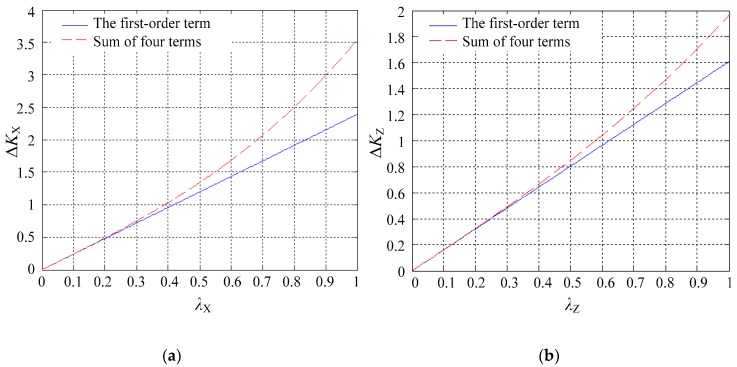
First-order term and the sum of four terms of (**a**) Δ*K*_X_ and (**b**)Δ*K*_Z_ if the displacements occur along the single-axis direction.

Figure 4 compares the first-order term and the sum of the four terms of dimensionless eccentric displacements in the case of the displacement in the single-axis direction. First, if the displacement of the ball occurs in the *X* direction (*λ*_X_ ≠ 0 and *λ*_Y_ = *λ*_Z_ = 0), the relative error between Δ*K*_X_ and its first-order term is 0.48% and 1.88% at *λ*_X_ = 0.1 and 0.2, respectively (Figure 4a). Second, if the displacement occurs in the *Y* direction (*λ*_Y_ ≠ 0 and *λ*_X_ = *λ*_Z_ = 0), the relative error between Δ*K*_Y_ and its first-order term is the same as that of the *X* direction and is therefore not shown. Third, if the displacement occurs in the *Z* direction (*λ*_Z_ ≠ 0 and *λ*_X_ = *λ*_Y_ = 0), the relative error between Δ*K*_Z_ and its first-order term is 0.22% and 0.88% at *λ*_Z_ = 0.1 and 0.2, respectively (Figure 4b).

**Figure 5:**
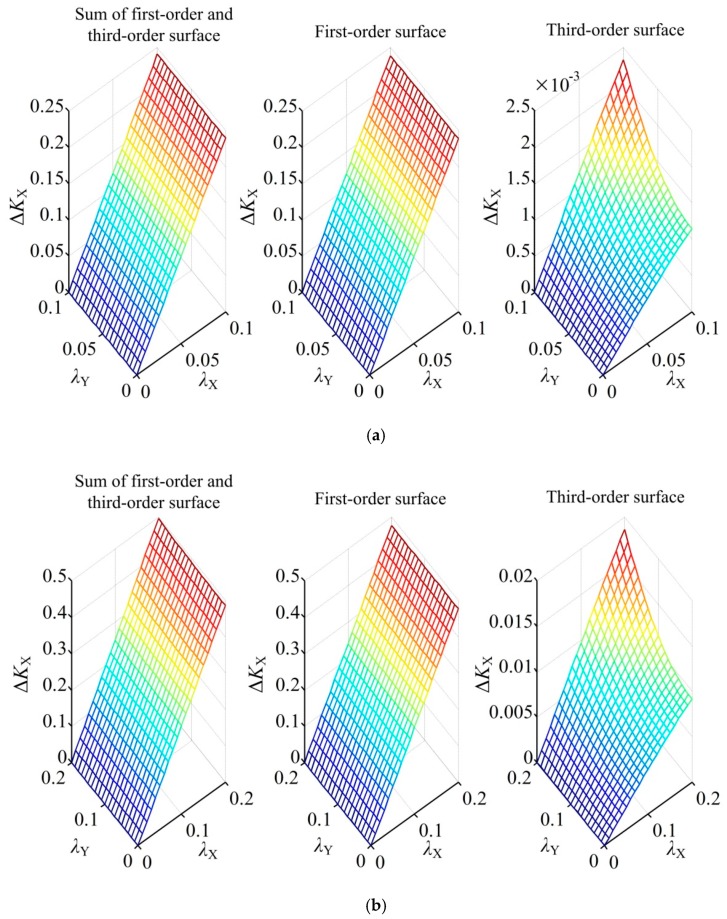
Sum of four terms, first-order term and third-order terms of Δ*K*_X_ with (**a**) *ρ* = 0.1 and (**b**) *ρ* = 0.2 if the displacements occur along the non-axis direction.

The complex case is that the ball has a small displacement in all three axis directions, assuming that the eccentricity of the ball $\rho =\sqrt{\lambda_{X}{}^{2}+\lambda_{Y}{}^{2}+\lambda_{Z}{}^{2}}$ is within the range of 0 ≤ *ρ* ≤ 1. Figure 5 presents the comparison between Δ*K*_X_ and its first-order term at *ρ* = 0.1 and *ρ* = 0.2, respectively. The difference between Δ*K*_X_ and its first-order term (i.e., third-order terms) is also included. Two observations can be made. First, Δ*K*_X_ is roughly equal to its first-order term on the condition of a small eccentric displacement of the ball. Specifically, the relative error between Δ*K*_Z_ and its first-order term is less than 1% and 4% at *ρ* = 0.1 and 0.2, respectively. Second, Δ*K*_X_ exhibits a linear dependence on *λ*_X_, whereas the effect of *λ*_Y_ and *λ*_Z_ on Δ*K*_X_ is negligible. Similarly, the corresponding conclusions for Δ*K*_Y_ and Δ*K*_Z_ can be inferred, respectively.

The above discussions indicate that the third-order terms of the eccentric displacements have little influence on the differential capacitance of the sensor if the eccentricity of the ball *ρ* ≤ 0.2. In addition, it is reasonable to use a linear function to describe the relationship between the differential capacitance and the spatially eccentric displacement component. Hence, if the eccentric displacement components *δ*_X_, *δ*_Y_ and *δ*_Z_ vary within a small range, Equations (17)–(19) can be rewritten in the following form:

(23) $$\Delta C_{X}=\frac{2.3896\varepsilon R_{0}{}^{2}}{e_{0}{}^{2}}\delta_{X},$$

(24) $$\Delta C_{Y}=\frac{2.3896\varepsilon R_{0}{}^{2}}{e_{0}{}^{2}}\delta_{Y},$$

(25) $$\Delta C_{Z}=\frac{1.6040\varepsilon R_{0}{}^{2}}{e_{0}{}^{2}}\delta_{Z}.$$

## 4. Simulation Setup

**Figure 6:**
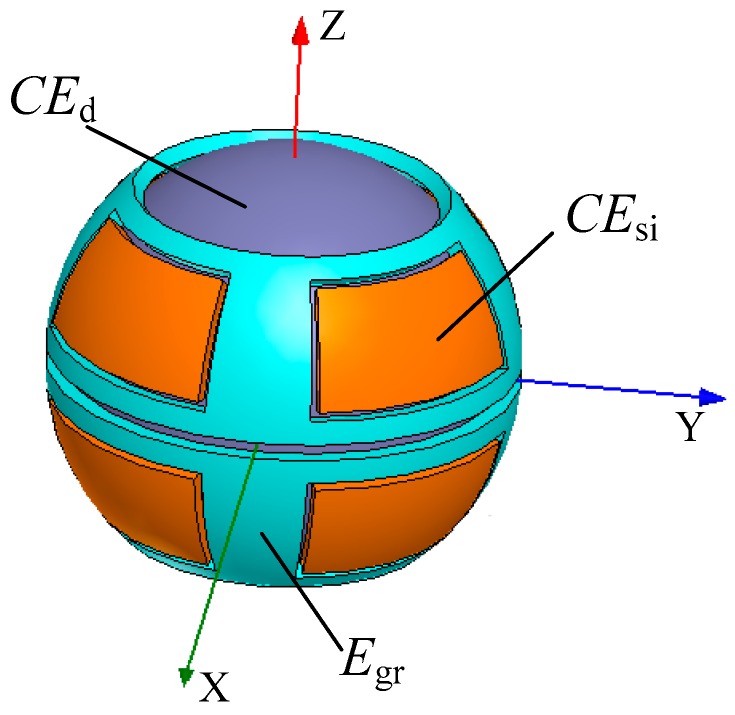
Simulation model of the SDCS with guard rings.

In practical applications, capacitive sensor plates have a certain thickness and a limited structure size. There are many charges unevenly distributed on each surface, and the density of the charges is much greater at the edge or tip end. Moreover, the electric field lines at the edge of the plate diverge outwards, resulting in the “fringe effect”. Therefore, the capacitance of the capacitive sensor is inevitably affected by the fringe effect [17]. The fringe effect not only decreases the sensitivity of capacitive sensor, but also generates nonlinear output which affects measuring accuracy. To reduce the errors caused by the fringe effect, equipotential guard rings are widely used in the design of capacitive sensors with spacing variation [18]. Therefore, guard rings (*E*_gr_ in Figure 6) for the proposed SDCS were designed on the periphery of the spherical capacitive plates. The radius of both inner and outer surfaces was equal to that of the spherical capacitive plates.

Ansoft Maxwell software was used to calculate the capacitance value at different eccentric displacements for the SDCS with and without guard rings. The spherical capacitive plates had an inner radius of 25.5 mm and a thickness of 2 mm. The radius of the ball was 25 mm, and other structural parameters of the SDCS are presented in Table 2. Abrasion-resistant material was used to fill in the gap between the ball and the spherical capacitive plates. However, air exited through the gap due to the assembly clearance. In order to simplify the simulation model and theoretical calculation, air was adopted as the dielectric. The permittivity of the air was 1.00053. The displacement varied from −50 μm to 50 μm, with a step of 2 μm.

## 5. Results and Discussions

### 5.1. Effect of Guard Rings

**Figure 7:**
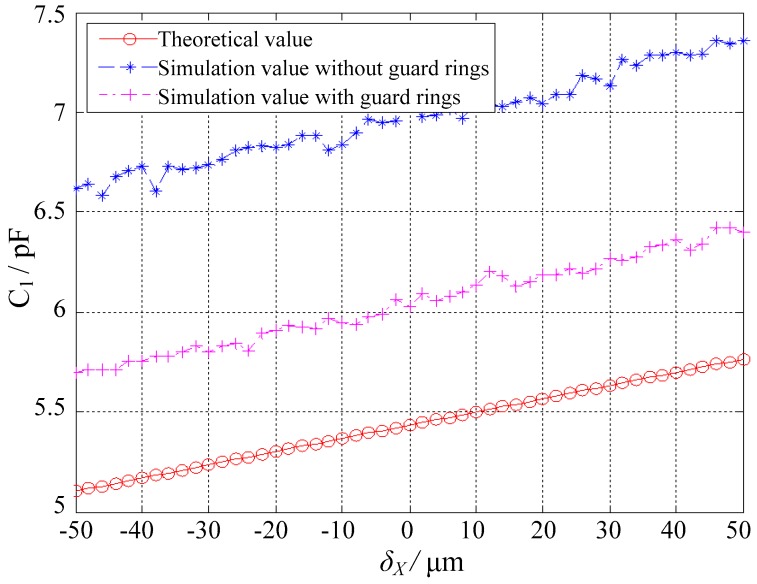
Effect of the guard rings on the capacitance value of the spherical capacitor *C*_1_.

Figure 7 presents the simulated capacitance values of the spherical capacitor *C*_1_ at different eccentric displacements, along with the theoretical results. Essential comparisons were made between the cases with and without guard rings. Two observations were made. First, the simulation capacitance values were larger than the theoretical values, which were mainly attributed to the fringe effect. This is consistent with Weng and Jin [19], that is, the fringe effect could lead to an increase in capacitance. Second, the increased capacitance was effectively reduced with guard rings, suggesting that the fringe effect could be weakened by deploying guard rings.

### 5.2. Dependence of the Differential Capacitances on the Eccentric Displacements

In this section, the dependence of the differential capacitances on the eccentric displacements is investigated in detail. To facilitate the discussion, the spatially eccentric displacement of the ball is divided into two categories based on the eccentric directions, namely, one along the single-axis direction and the other along the non-axis direction.

**Figure 8:**
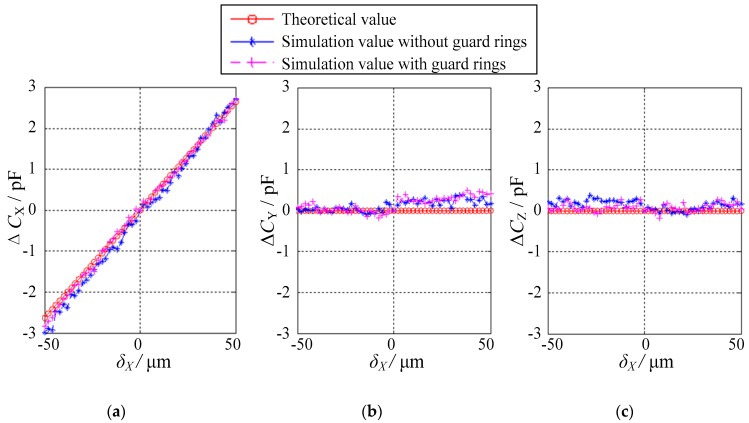
Relationship between the differential capacitance values and the displacements in the case of *δ*_Y_ = *δ*_Z_ = 0: (**a**) Δ*C*_X_, (**b**) Δ*C*_Y_ and (**c**) Δ*C*_Z_.

The simplest case is that the ball exhibits eccentric displacements in one of three orthogonal axes (i.e., *X*, *Y* or *Z* direction). The authors have selected the eccentric displacements along the *X* direction *δ*_X_ as an example. *δ*_X_ varies from −50 μm to 50 μm, with a step of 2 μm. Evidently, the eccentricity of the ball is less than 0.1. Figure 8 presents the dependence of the differential capacitance Δ*C*_X_, Δ*C*_Y_ and Δ*C*_Z_ on the displacements along the *X* direction *δ*_X_. Three observations can be made. First, the variation of the simulated values of Δ*C*_X_, Δ*C*_Y_ and Δ*C*_Z_ exhibits similar trends with that of the theoretical values. In other words, the simulation values are consistent with the theoretical values. Second, the differential capacitance Δ*C*_X_ exhibits a linear relationship with the displacement *δ*_X_, suggesting a good linearity of the proposed sensor. Third, the simulated values of Δ*C*_Y_ or Δ*C*_Z_ are close to the theoretical values, that is, 0 pF over the range of −50 μm ≤ *δ*_X_ ≤ 50 μm. This indicates that no displacement of the ball occurs along the *Y* or *Z* direction. The simulated values exhibit small deviations of 0.5 pF in Δ*C*_Y_ and 0.4 pF in Δ*C*_Z_ from the theoretical values, which may be ascribed to the simulation errors.

**Figure 9:**
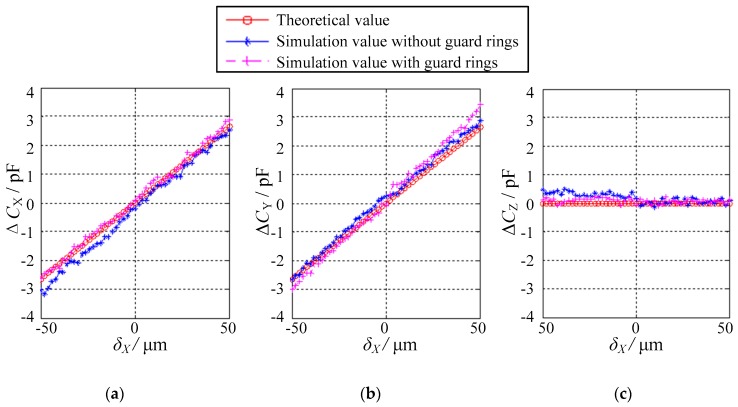
Relationship between the differential capacitance values and the displacements in the case of *δ*_X_ = *δ*_Y_ and *δ*_Z_ = 0: (**a**) Δ*C*_X_, (**b**) Δ*C*_Y_ and (**c**) Δ*C*_Z_.

**Figure 10:**
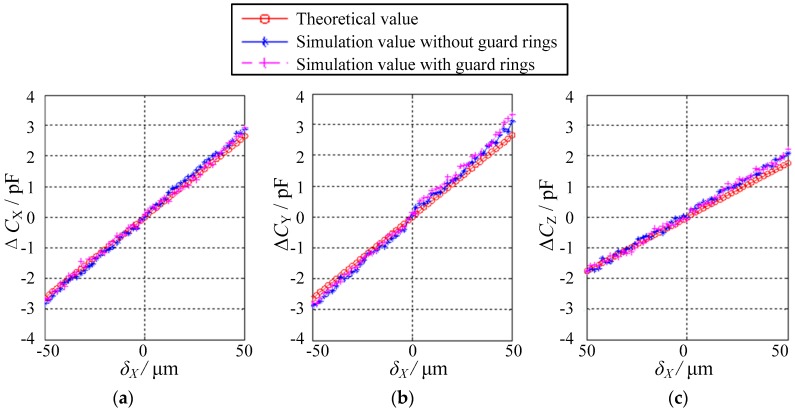
Relationship between the differential capacitance values and the displacements in the case of *δ*_X_ = *δ*_Y_ = *δ*_Z_: (**a**) Δ*C*_X_, (**b**) Δ*C*_Y_ and (**c**) Δ*C*_Z_.

Figure 9 and Figure 10 present the simulated values of the differential capacitances Δ*C*_X_, Δ*C*_Y_, and Δ*C*_Z_ on the condition that the eccentric displacements are along non-axis directions, the former being along the direction of *X* = *Y* in the *XOY* plane (*δ*_X_ = *δ*_Y_ and *δ*_Z_ = 0) and the latter being along the direction of *X* = *Y* = Z in three-dimensional (3D) space (*δ*_X_ = *δ*_Y_ = *δ*_Z_). The maximum eccentricity is 0.14 for the eccentric displacements in the *XOY* plane and 0.17 for those in space, respectively. Several observations can be made. First, the simulated values of Δ*C*_X_, Δ*C*_Y_ and Δ*C*_Z_ are roughly equal to their theoretical values. Second, if the eccentric displacements are along the direction of *X* = *Y* in the *XOY* plane, both Δ*C*_X_ and Δ*C*_Y_ exhibit a linear correlation with *δ_x_*, whereas the variation of Δ*C*_Z_ over the range of −50 μm ≤ *δ*_X_ ≤ 50 μm approximately remains unchanged (Figure 9). Third, if the eccentric displacements are along the direction of *X* = *Y* = Z in 3D space, all the differential capacitances Δ*C*_X_, Δ*C*_Y_ and Δ*C*_Z_ show a linear relationship with *δ*_X_ (Figure 10). Finally, the gradient of Δ*C*_Z_ is lower than that of Δ*C*_X_ and Δ*C*_Y_. This indicates that the sensitivity along the *Z* direction is lower than that along the *X* and *Y* directions, which is consistent with Equations (23)–(25).

In summary, it can be inferred from the simulation result that the eccentric displacement component along the *X*, *Y* or *Z* direction has a good linear relationship with corresponding differential capacitance, provided that the eccentricity of the ball is small. Furthermore, due to the symmetrically spatial layout of the spherical capacitive plates and the differential calculation for the capacitance of the spherical capacitors, additional capacitance caused by the fringe effect can be effectively reduced.

## 6. Conclusions

This work proposes a novel method for the micro-clearance measurement of a precision spherical joint based on an SDCS. The configuration of the SDCS was first designed and a mathematical model of the proposed measurement method was then deduced. Finally, a simulation using Ansoft Maxwell software was conducted to verify the feasibility of the proposed method. The following conclusions can be made.

(1) The developed SDCS included a ball and eight spherical capacitive plates that were fixed onto the inner side of the socket. The ball was deployed as an excitation electrode, whereas each of the eight capacitive plates was used as a sensing electrode. As such, each capacitive plate and the ball formed a capacitor. The eccentric displacements of the ball were obtained by detecting the capacitance of eight spherical capacitors.

(2) A mathematical model of the differential capacitances and eccentric displacements was deduced. Due to the symmetrically spatial layout of the spherical capacitive plates and the differential calculation for the capacitance of the spherical capacitors, the even-order terms of dimensionless eccentric displacements of the ball in the socket were eliminated. Furthermore, if the eccentricity of the ball was less than 0.2, the third-order terms of the eccentric displacements had little effect on the differential capacitance of the sensor and were therefore neglected. As such, the differential capacitances Δ*C*_X_, Δ*C*_Y_ and Δ*C*_Z_ exhibited a linear correlation with the eccentric displacement components *δ*_X_, *δ*_Y_ and *δ*_Z_, respectively.

(3) The simulated values approximately coincided with the theoretical values, showing a linear correlation between the differential capacitances Δ*C*_X_, Δ*C*_Y_ and Δ*C*_Z_ and the eccentric displacements. This suggests the feasibility of the proposed method for the clearance measurement of a spherical joint. Moreover, the simulated values with guard rings are closer to the theoretical values than those without guard rings. This indicates that the capacitance derivation caused by the fringe effect can be effectively reduced by employing guard rings, that is, the measurement accuracy can be improved by deploying guard rings.

Thus, this work presents analytical and numerical investigations on a capacitive sensor for the clearance measurement of spherical joints. Further studies are still required on experimental investigations about the performance of an actual sensor. Moreover, the sensor performance may be affected by geometrical tolerances (e.g., spherical tolerances of the ball and symmetrical tolerances of the capacitive plates). Extensive studies are required to assess a specification on needed tolerances and to compare it with typical spherical joint tolerances. Finally, mathematical deductions using Pade approximation and Schwarz–Chistoffel mapping may be also interesting objectives of further studies that aim at improving the prediction or theoretical calculation of sensor performance.

## Figures and Tables

**Table 1:** Spherical capacitors included in three pairs of differential capacitance units along *X*, *Y* and *Z* directions, respectively.

Unit Pair	Capacitors for Unit 1	Capacitors for Unit 2
*C*_up-*X*_	*C*_1_, *C*_4_, *C*_5_ and *C*_8_	*C*_2_, *C*_3_, *C*_6_ and *C*_7_
*C*_up-*Y*_	*C*_1_, *C*_2_, *C*_5_ and *C*_6_	*C*_3_, *C*_4_, *C*_7_ and *C*_8_
*C*_up-*Z*_	*C*_1_, *C*_2_, *C*_3_ and *C*_4_	*C*_5_, *C*_6_, *C*_7_ and *C*_8_

**Table 2:** Simulation parameters of the SDCS.

Parameters	Values
The angle of spherical capacitive plates in the longitude direction *ζ*	π/6
The angle of spherical capacitive plates in the latitude direction *ξ*	π/3
The angle of the clearance between the guard ring *η*	π/90

## References

[1] Robertson A.P., Slocum A.H. (2006). Measurement and characterization of precision spherical joints. Precis. Eng..

[2] Zhu J., Ting K.L. (2000). Uncertainty analysis of planar and spatial robots with joint clearances. Mech. Mach. Theory.

[3] Han Y., Zhong C., Zhu X., Zhe J. (2018). Online monitoring of dynamic tip clearance of turbine blades in high temperature environments. Meas. Sci. Technol..

[4] Garcia I., Przysowa R., Amorebieta J., Zubia J. (2016). Tip-clearance measurement in the first stage of the compressor of an aircraft engine. Sensors.

[5] Zhang J., Duan F., Niu G., Jiang J., Li J. (2017). A blade tip timing method based on a microwave sensor. Sensors.

[6] Jamia N., Friswell M.I., El-Borgi S., Fernandes R. (2018). Simulating eddy current sensor outputs for blade tip timing. Adv. Mech. Eng..

[7] Lawson C.P., Ivey P.C. (2005). Tubomachinery blade vibration amplitude measurement through tip timing with capacitance tip clearance probes. Sens. Actuators A Phys..

[8] Endemano A., Desmulliez M.P.Y., Dunnigan M. (2002). System level simulation of a double stator wobble electrostatic micromotor. Sens. Actuators A Phys..

[9] Han F.T., Gao Z.Y., Li D.M., Wang Y.L. (2005). Nonlinear compensation of active electrostatic bearings supporting a spherical rotor. Sens. Actuators A Phys..

[10] Han F.T., Gao Z.Y., Wang Y.L. (2002). Modelling and linearization for long-range spherical gap measurement. Chin. J. Sci. Instrum..

[11] Gao Z.Y. (2004). Electrostatically Suspended Gyroscope Technology.

[12] Hill D.A., Letendre T., Mills H.A. Embedded, real-time DSP control of an electrostatically suspended gyroscope. Proceedings of the 2004 American Control Conference.

[13] Liu J.H., Wang H., Chang K., Li X., Wang Q.L. Design and test of displacement transducer for an electrode-insulated electrostatically suspended gyroscope. Proceedings of the 2015 IEEE International Conference on Applied Superconductivity and Electromagnetic Devices.

[14] Han F.T., Wu Q.P., Gao Z.Y. (2006). Initial levitation of an electrostatic bearing system without bias. Sens. Actuators A Phys..

[15] Baxter L.K. (1997). Capacitive Sensors: Design and Applications.

[16] Heerens W.C. (1986). Application of capacitance techniques in sensor design. J. Phys. E Sci. Instrum..

[17] Wang W., Wen Y.H., Yu J.P., Chen Z. (2011). Impact of fringe effect on measuring accuracy of planar capacitive sensors. Sens. Lett..

[18] Ma Y.Z., Yu Y.X., Wang X.H. (2013). Key technique for inner hole diameter measure with capacitive probe. Appl. Mech. Mater..

[19] Weng C.W., Jin A.K. (1980). Effect of fringing fields on the capacitance of circular microsrip disk. IEEE Trans. Microw. Theory.

